# Altered hypoxia-induced cellular responses and inflammatory profile in lung fibroblasts from COPD patients compared to control subjects

**DOI:** 10.1186/s12931-024-02907-x

**Published:** 2024-07-16

**Authors:** Ryde Martin, Marek Nora, Löfdahl Anna, Pekny Olivia, Bjermer Leif, Westergren-Thorsson Gunilla, Tufvesson Ellen, Larsson-Callerfelt Anna-Karin

**Affiliations:** 1 Lung Biology, Department of Experimental Medical Science, Faculty of Medicine, Lund University, Lund, Sweden; 2 Respiratory Medicine, Allergology and Palliative Medicine, Department of Clinical Sciences Lund, Faculty of Medicine, Lund University, Lund, Sweden

**Keywords:** Hypoxia, Lung fibroblasts, COPD, Gene expression, Inflammation, Remodelling, Oxidative stress, ER stress

## Abstract

**Background:**

Chronic obstructive pulmonary disease (COPD) is a heterogeneous disease characterized by chronic bronchitis, emphysema and vascular remodelling. The disease is associated with hypoxia, inflammation and oxidative stress. Lung fibroblasts are important cells in remodelling processes in COPD, as main producers of extracellular matrix proteins but also in synthesis of growth factors and inflammatory mediators.

**Methods:**

In this study we aimed to investigate if there are differences in how primary distal lung fibroblasts obtained from COPD patients and healthy subjects respond to hypoxia (1% O_2_) and pro-fibrotic stimuli with TGF-β_1_ (10 ng/mL). Genes and proteins associated with oxidative stress, endoplasmic reticulum stress, remodelling and inflammation were analysed with RT-qPCR and ELISA.

**Results:**

Hypoxia induced differences in expression of genes involved in oxidative stress (SOD3 and HIF-1α), ER stress (IRE1, PARK and ATF6), apoptosis (c-Jun and Bcl2) and remodelling (5HTR2B, Collagen7 and VEGFR2) in lung fibroblasts from COPD subjects compared to control subjects, where COPD fibroblasts were in general less responsive. The release of VEGF-C was increased after hypoxia, whereas TGF-β significantly reduced the VEGF response to hypoxia and the release of HGF. COPD fibroblasts had a higher release of IL-6, IL-8, MCP-1 and PGE_2_ compared to lung fibroblasts from control subjects. The release of inflammatory mediators was less affected by hypoxia, whereas TGFβ1 induced differences in inflammatory profile between fibroblasts from COPD and control subjects.

**Conclusion:**

These results suggest that there is an alteration of gene regulation of various stress responses and remodelling associated mediator release that is related to COPD and hypoxia, where fibroblasts from COPD patients have a deficient response.

**Supplementary Information:**

The online version contains supplementary material available at 10.1186/s12931-024-02907-x.

## Background

COPD is a severe heterogeneous lung disease with no cures or effective treatments available. Causes of the disease are related to exposure to smoke and air pollutions [1]. The disease is characterised by airflow limitations linked to chronic inflammation, fibrosis around the small airways and emphysema. Lack of sufficient oxygen supply in the lung results in hypoxic milieus which trigger cellular activity and pathological remodelling processes [2]. These events contribute to a decline in effective oxygen exchange in the alveoli and to progression of the disease. Hypoxia has mostly been studied in different cancer diseases and less in detail in ongoing remodelling processes in chronic lung diseases, highlighting the need of more research into this area. The transcriptional activator hypoxia-inducible factor (HIF) is induced by hypoxia and a modulator of oxygen homeostasis. Activation of HIF induces expression of matrix metalloproteinases and vascular endothelial factor (VEGF) to increase oxygen delivery by promoting vascular remodelling and angiogenesis [3].

Pulmonary vascular remodelling is common in COPD [4] and comorbidities in cardiovascular disease have negative impacts on COPD prognosis [5]. Airflow obstructions and fibrosis in small airways and emphysema with destruction of alveolar capillaries and epithelial cells result in decreased oxygen transport and alveolar hypoxia [2]. We have previously shown that VEGF is synthesised by primary human lung fibroblasts and induced by transforming growth factor (TGF)-β_1_ and prostaglandins [6] and that COPD patients have an altered fibroblast phenotype [7]. Fibroblasts are key players in regulating the homeostasis of extracellular matrix (ECM) but also in pathological remodelling processes by constituting a rich source of cytokines and growth factors and responding to profibrotic stimuli [8]. Increased VEGF expression is associated with bronchial angiogenesis that inversely correlated with lung function in COPD patients [9]. Other important pathological changes appear in COPD, such as alterations in gene expressions related to ER stress and unfolded protein responses (UPR) [10], which have been linked to oxidative stress and mitochondrial dysfunction [2].

The aim of this project was to assess if exposure to a hypoxic milieu induces responses in distally derived lung fibroblasts that mimic pathological changes, involving oxidative stress and ER stress, inflammation and remodelling, as hypoxia is implicated to be a driver for COPD progression, and if there were differences in inflammatory response to hypoxia and profibrotic stimuli between the lung fibroblasts obtained from healthy subjects and COPD patients. Investigated genes were associated with ER stress and UPR (IRE1, PERK, ATF6 and CHOP, PSMA1, PSMB6 and PSMD11), oxidative stress (Nrf2, OXR1, SOD3), metabolic or mitochondrial stress responses (PINK1 and Parkin), inflammation (PTGS2) and hypoxia (HIF-1α). Other genes of interest were the VEGF receptors 2 and 3 linked to angiogenesis and vascular remodelling, basement membrane collagen VII linked to airway remodelling responses in COPD [11] and serotonin receptor 5-HTR2B associated with lung fibrosis [12]. We hypothesized that differences observed in gene and protein expression, related to cellular stress, inflammation and remodelling in COPD, are induced by hypoxia exposure in healthy fibroblasts which may give an increased understanding how hypoxia could be an initiator or driver of pathological alterations observed in COPD. Changes in measured markers related to hypoxia exposure may be useful as biomarkers of disease onset or progression. We also hypothesized that the response to hypoxia and profibrotic stimuli would be different between lung fibroblasts obtained from healthy subjects and COPD patients. Our findings indicate that hypoxia and profibrotic stimuli induced cellular responses and altered inflammatory profile with fibroblasts from COPD patients being less responsive compared to control subjects.

## Materials & methods

### Patient material

Primary distally derived human lung fibroblasts were obtained after isolation from the alveolar regions from either explants from healthy subjects intended to be used for lung transplantation (donor lungs), or patients with very severe COPD (GOLD IV) subjected to lung transplantation. In addition, lung resection tissue was obtained from distal areas of tumour sites from smokers and patients with well-characterised moderate COPD (GOLD II), see Table 1 for detailed info. There was no information available if the COPD patients were diagnosed with pulmonary hypertension. The study was conducted according to the guidelines of the Declaration of Helsinki and approved by the Regional Ethical Review Board in Lund, Sweden (FEK 2015/891) and the Swedish Research Ethical Committee in Gothenburg (FEK675–12/2012) and performed in accordance with guidelines approved by the ethical committees. Written informed consent was obtained from the closest relatives of healthy lung donors and from patients included in the study.

**Table 1:** Information about patient material

Patient	Disease stage	Sex	Age	Smoking history	Pack-years	FEV_1_	FEV_1_/FCV (%)	DLCO (%)
1	Healthy	M	62	Never	-	N/A	N/A	N/A
2	Healthy	M	56	Never	-	N/A	N/A	N/A
3	Healthy	M	62	Former	N/A	N/A	N/A	N/A
4	Healthy	F	65	Never	N/A	N/A	N/A	N/A
5	Healthy	F	36	Former	18	1.78	74	64
6	Healthy	F	65	Former	20	2.5	74	96
7	Healthy	M	71	Former	25	2.69	74	62
8	GOLD II	F	68	Former	31	2.27	62	91
9	GOLD II	M	67	Former	51	2.18	63	56
10	GOLD II	M	51	Former	32	3.3	57	82
11	GOLD II	F	73	Former	41	1.54	70	81
12	GOLD IV	M	59	Former	40	0.5	N/A	N/A
13	GOLD IV	F	63	Former	35	0.6	40	45
14	GOLD IV	F	66	Former	30	1.27	45	27

N/A: not available

### Culture of primary human lung fibroblasts

Distally-derived lung fibroblasts were obtained from alveolar parenchymal specimens. The tissue was collected 2–3 cm from the pleura in the lower lobes, i.e. from the same location as where transbronchial biopsies were taken. Vessels and small airways were removed from the peripheral lung tissues and the remaining tissues were chopped into small pieces. Parenchymal pieces from explants were allowed to adhere to the plastic of cell culture flasks for 4 h before addition of Dulbecco’s modified eagle medium (DMEM) GlutaMAX™ (Gibco™, Thermo Scientific, Cat# 10,566,016, Waltham, MA, USA) supplemented with 10% FetalClone III serum (FCIII, HyClone Laboratories, Cat# SH30109.03, Marlborough, MA, USA), 1 mM sodium pyruvate (Stock 100 mM, Sigma-Aldrich, Cat# S8636, Darmstadt, Germany), 2.5 mg/mL Amphotericin B solution (AmpB, Stock 250 mg/mL, Sigma-Aldrich, Cat# A2942, Darmstadt, Germany) and 0.25 mg/mL Gentamycin (Stock 50 mg/mL, Thermo Fisher, Cat# 15,750,037, Waltham, MA, USA) (complete medium) in a humidified incubator at 37 °C and 10% CO_2_. The culture flasks were then kept in cell culture medium (DMEM) in 37 °C cell incubators until outgrowth of fibroblasts were observed. Parenchymal fibroblasts were then referred to as distal lung fibroblasts [13]. The primary human distally-derived lung fibroblasts were cultured in Dulbecco’s modified eagle medium (DMEM) GlutaMAX™ (Gibco™, Thermo Scientific, Cat# 10,566,016, Waltham, MA, USA) supplemented with 10% FetalClone III serum (FCIII, HyClone Laboratories, Cat# SH30109.03, Marlborough, MA, USA), 1 mM sodium pyruvate (Stock 100 mM, Sigma-Aldrich, Cat# S8636, Darmstadt, Germany), 2.5 mg/mL Amphotericin B solution (AmpB, Stock 250 mg/mL, Sigma-Aldrich, Cat# A2942, Darmstadt, Germany) and 0.25 mg/mL Gentamycin (Stock 50 mg/mL, Thermo Fisher, Cat# 15,750,037, Waltham, MA, USA) (complete medium) in a humidified incubator at 37 °C and 10% CO_2_. Cells were passaged upon confluency and cell culture medium was exchanged every 3 days.

### Hypoxia exposure of primary human lung fibroblasts

Cells were seeded at a density of 15–30 × 10^4^ cells/well in 2 mL complete medium in 6-well plates (Nunclon Delta Surface, Thermo Scientific Waltham, MA, United States) and cultured at 37 °C with 10% CO_2_ until approximately 70–80% confluency. Cells were then starved with supplemented DMEM GlutaMAX™ medium containing 0.4% FC III in a humidified incubator at 37 °C and 10% CO_2_ for 2 h. Medium was then changed to new starvation medium with or without addition of 10 ng/mL of profibrotic TGF-β1 (R&D Systems, Cat# 240-B, Minneapolis, MN) 10 min before hypoxia or normoxia exposure. Plates for hypoxia exposure were placed in a hypoxic chamber [model CO2-O2 UNIT-BL (0–20, 1–95), Okolab, Ottaviano, NA, Italy] set to 37 °C, 90% humidity, 1% O_2_ and 10% CO_2_ and the other plates were placed in a humidified incubator with normoxic standard culture conditions (37 °C, 21% O_2_, 10% CO_2_) for either 4–24 h, as previously described in Berggren-Nylund et al [14]. After the exposure, the supernatant was immediately collected for further analysis and the cell layer was collected to either mRNA analysis or protein analysis and stored at -80 °C. All the exposure studies were performed in passage 3–6.

### Total protein amount in cell cultures

To collect cells for protein analysis, 100 mL NP-40 lysis buffer (Thermo Fisher, Cat# FNN0021, Waltham, MA, USA) containing 1% protease inhibitor (Sigma-Aldrich, Cat# P8340, Darmstadt, Germany) was added to the cells. The cells were scraped off and the samples were centrifuged at 10.000 rcf at 4 °C for 10 min. The supernatant was collected and stored at -80 °C. Protein concentrations were determined with Pierce™ BCA Protein Assay Kit (Thermo Fisher Scientific, Cat# 23,225, Waltham, MA, USA) following manufacturer’s instructions.

### Gene expression analysis

Ice-cold RLT buffer (0.3 mL) with 1% β-mercaptoethanol was added to the cell layer and total RNA was purified using RNeasy Mini Kit (Qiagen, Cat# 74,106, Hilden, Germany). Cells were then scraped off and a syringe was used to homogenise the sample. Samples were stored at -80 °C until purification. To increase yield of RNA purification using RNeasy Mini Kit, the centrifugation times were increased to 60 s for all washing steps and 3 min for the last washing step. Briefly, RNA samples of the same conditions (normoxia versus hypoxia) were pooled. DNA was removed with RNase-free DNase Set (Qiagen, Cat#79,256, Hilden, Germany). RNA concentration was measured with a NanoDrop. cDNA was synthesised using iScript™cDNA Synthesis Kit (Bio-Rad, Cat# 170–8890, Hercules, CA, USA). The reaction mix was prepared as described in the manufacturer’s instructions and scaled for RNA amounts > 1000 ng. qPCR reaction mixes were prepared using iTaq™ Universal SYBR^®^ Green Supermix (Bio-Rad, Cat# 1,725,124, Hercules, CA, USA) following the manufacturer’s instructions. 25 ng cDNA was added into each well containing 300 nM of each primer. Genes analysed were: DNA damage-inducible transcript 3 protein (CHOP), Nuclear factor erythroid 2-related factor 2 (NRF2), Serine/threonine-protein kinase/endoribonuclease (IRE1), Eukaryotic translation initiation factor 2-alpha kinase 3 (PERK), Cyclic AMP-dependent transcription factor ATF-6 alpha (ATF6), Proteasome subunit alpha type-1 (PSMA1) and beta type-6 (PSMAB6), 26 S proteasome non-ATPase regulatory subunit 11 (PSMD11), Oxidation resistance protein 1 (OXR1), Apoptosis regulator Bcl-2, VEGFR1, VEGFR2, VEGFR3, 5-HTR2B, Collagen 7, HIF-1α, E3 ubiquitin-protein ligase parkin (PARK), Serine/threonine-protein kinase (PINK1), Extracellular superoxide dismutase (SOD3), Transcription factor AP-1 oncogene (c-Jun), Prostaglandin G/H synthase 2 (PTGS2). The housekeeping genes GAPDH, 18 S, β-actin were analysed. Data indicated that GADPH was unstable in the hypoxia experiments and was therefore excluded as housekeeping gene. β-actin and 18 S were both stable during the experimental settings and a geometric mean of these two housekeeping genes was used for all the gene expression analysis. Data is presented as 2^dCT (Ct housekeeping gene – Ct gene of interest). The primer sequences are shown in Additional data 1 Table S1. Ct values of 38 or higher were excluded from the data.

### Measurements of cell viability

Cells were cultured in 24-well cell culturing plates and incubated for 4 h, 24–72 h at 37⁰C, 10% CO_2_ in either hypoxia or normoxia. Cell medium was collected and analysed for cytotoxicity using Lactate Dehydrogenase Activity Assay Kit (Roche, Cat. No. 11 644 739 001, Basel, Switzerland) and the manufacturer’s instructions were followed. Cells treated with 1% Triton-X100 were used as a positive control for cell death. The cell culture plates with cells prepared for the LDH assay were further analysed for metabolic activity using a Water-soluble tetrazolium salt 1 (WST-1) test (Roche, 11 644 807 001). Remnants of cell medium were removed and 10% WST-1 medium/well were added. Plates continued exposure to hypoxic or normoxic conditions for 1 h. Absorbance of cell culture medium was measured in a microplate reader at 440 nm and 620 nm (as reference).

### Measurements of growth factors and inflammatory mediators

The release of several cytokines and growth factors were analysed in cell culture medium from cells exposed to 24 h normoxic or hypoxic conditions with Bio-Plex Pro Human Cytokine assay according to the manufacturer’s instructions (Bio-Rad, Cat# 12,007,283, Hercules, CA, USA). The following cytokines were analysed: monocyte chemotactic protein-1 (MCP-1), interleukin (IL)-6, IL-8, fibroblast growth factor-basic (FGF-basic, also known as FGF-2), hepatocyte growth factor (HGF), Regulated upon Activation, Normal T Cell Expressed and Secreted (RANTES) and tumour necrosis factor (TNF)-alpha. The calibration curves were fitted using a five-point regression model and the results were evaluated in the Bio-Plex Manager Software 6.0 (Bio-Rad). The lower limit of quantification was 1.4 (IL-6), 3.0 (IL-8), 2.6 (MCP-1), 0.12 (RANTES), 1.9 (FGF basic) and 7.6 (HGF) pg/mL, respectively.

VEGF-A and VEGF-C were analysed in the cell culture medium using Human VEGF-A Quantikine ELISA kit and Human VEGF-C Quantikine ELISA kit DVC00 (both from R&D Systems, Minneapolis, MN, United States), according to manufacturer’s instructions. The absorption was measured at 450 and 570 nm (as reference). The lower detection limit of quantification was 7.8 pg/mL for VEGF-A and 55 pg/mL for VEGF-C. Prostacyclin and prostaglandin E_2_ (PGE_2_) were analysed using 6-keto Prostaglandin F_1α_ (a stable metabolite of prostacyclin) ELISA Kit (Cayman Chemical, Cat# 515,211, Ann Arbor, MI, USA) and PGE_2_ ELISA Kit – Monoclonal (Cayman Chemical, Cat# 514,010, Ann Arbor, MI, USA) following the manufacturer’s instructions. The absorbance was measured at 415 nm with a microplate reader. The assay detection limit for PGF_1α_ was 1.6 pg/mL and PGE_2_ was 7.8 pg/mL. Total amount of released mediator was normalized to total protein amount for each individual sample.

### Immunocytochemistry staining

Fibroblasts were cultured (10 000 cells/well) in chamber slides (ThermoFisher Scientific, 177,399) with four wells /slide. The slides were cultured for 24 h in either hypoxia or normoxia. The cells were fixed in 4% formaldehyde solution for 15 min at room temperature. Cells were washed in D-PBS and stored at 4⁰C. For HIF-1α staining, cells were permeabilized for 5 min with Triton-X100 0.2% in PBS. The following primary antibodies were used: HIF-1α (dilution 1:50, Biotin, ab81633), HIF-2α (dilution 1:100 Novus Biologicals, Bio-Techne, #NB100-132), 5HTR2B (dilution 1:300, Aviva System Biology, OAAF02801), VEGFR2 (dilution 1:200, Bio-rad, AHP-1327), VEGFR3 (dilution 1:200, Abcam, GR3217142-6) and negative control (Dako, X0903), all diluted in 1% BSA in TBS. Slides were incubated without light exposure at room temperature for 90 min. Slides were washed in TBS for 2 × 5 min and thereafter incubated for 45 min with the secondary antibody (dilution 1:200, anti-rabbit, Cy3 conjugated, Life technologies, A21428) supplemented (separately) with 4′,6-diamidino-2-phenylindole (DAPI; 1:500) for nuclear staining. Slides were washed for 2 × 5 min in TBS and mounted with fluorescence mounting medium (Dako). Slides were stored at 4⁰C until microscopy analysis. Fluorescence images were obtained with an automated slide scanner (Olympus VS-120 S, Olympus, VS120-L100-FL080, Hamburg, Germany) in the DAPI, Cy5 and FITC or Cy3 channel. The HIF-2α stainings were quantified with QuPath v0.4.4 [15] with which a threshold of 3000 maximum pixel intensity within the whole cell body (both cytoplasm and nucleus) was set to determine number of positive cells. The percentages of positive cells were then compared, and statistics were run with Graphpad Prism (repeated measures ANOVA for paired comparisons and two-way ANOVA for unpaired comparisons). Images were processed in OlyVia 2.9.1 (Olympus, Hamburg, Germany), by adjusting the signal intensity through gating to remove autofluorescence and background, and to allow for comparisons between slides. The same gating for the signal was applied to correlated images.

### Statistical analysis

All statistical analyses were performed using the software GraphPad Prism 9.3.1 (San Diego, USA). Data are presented as individual values with median. Statistical analyses were performed with one-way or two-way repeated measurements (RM) ANOVA followed by post-hoc test (Fisher’s LSD) using the software GraphPad Prism 9.1.2 (San Diego, USA). A p-value below 0.05 (*p* < 0.05) was statistically significant.

## Results

### Experimental settings of hypoxic conditions in healthy primary lung fibroblasts

A pilot study was first conducted in primary healthy distally-derived lung fibroblasts. Different oxygen concentrations (1%, 2% or 5% O_2_), serum concentrations (0.4%, 1% or 2%) in the culture medium and time points (4 h, 24 h, 48 h and 72 h) were evaluated for the settings for hypoxia exposures in primary healthy lung fibroblasts (Additional data file 2, Figure Supplement 1a-l). Based on these experiments, we set hypoxia exposure to 1% oxygen, exposure time to 4 h and 24 h and cell medium containing 0.4% serum for following experiments with primary lung fibroblasts.

### Effects of hypoxia on gene expression in distal lung fibroblasts from COPD and healthy

**Fig. 1:**
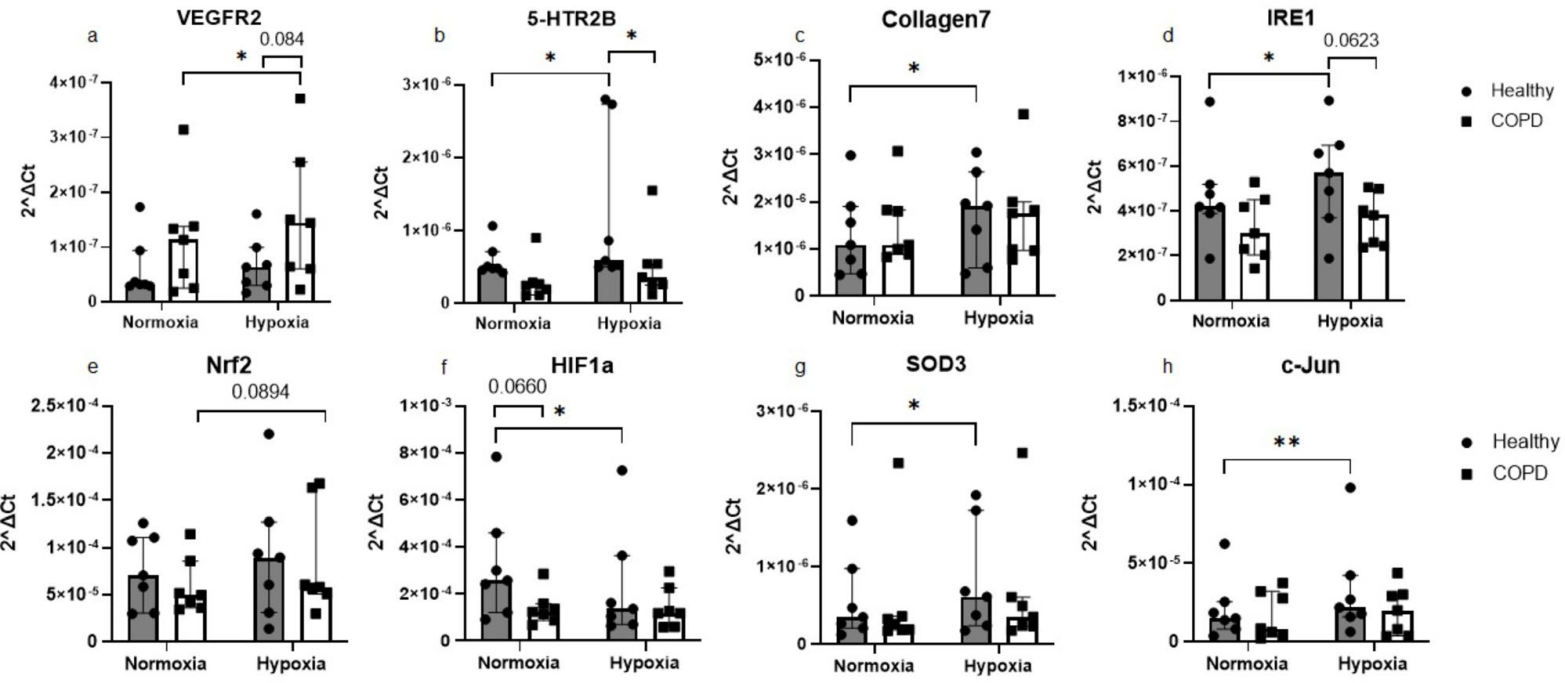
Effect of 4 h of hypoxia exposure on gene expression levels. Genes related to remodelling: VEGFR2 (**a**), 5-HTR2B (**b**) and Collagen7 **(c)**; cellular stress: IRE1 (**d**) and c-Jun (**h**), oxidative stress: Nrf2 (**e**), HIF-1α **(f)** and SOD3 (**g**) were measured by RT-qPCR in primary distal lung fibroblasts obtained from healthy subjects (*n* = 7) and COPD patients (*n* = 7) after 4 h of exposure to normoxic (21% O_2_) or hypoxic (1% O_2_) conditions. Beta-actin and 18 S were used as housekeeping genes. The data is presented as median with interquartile range. Ordinary two-way ANOVA or RM two-way ANOVA were used for unpaired and paired comparisons and the post-hoc test Fisher’s LSD was used for statistical analysis *p < 0.05, **p < 0.01

Alterations in gene expression after hypoxia exposure (1% O_2_) was investigated using RT-qPCR. As we could not detect any significant differences in gene expression between fibroblasts from non-smokers and former smokers or fibroblasts from COPD patients with GOLD II and GOLD IV, the gene expression results were grouped as healthy vs. COPD. After 4 h of hypoxia exposure there were increased expression of 5-HTR2B (*p* = 0.023, Fig. 1b), Collagen7 (*p* = 0.027, Fig. 1c), IRE1 (*p* = 0.019, Fig. 1d), SOD3 (*p* = 0.021, Fig. 1g), and c-Jun (*p* = 0.005, Fig. 1h) and a decrease in expression of HIF-1α (*p* = 0.036, Fig. 1f) in fibroblasts from control subjects, whereas there was an increase in expression of VEGFR2 (*p* = 0.035, Fig. 1a) in fibroblasts from COPD patients. We also investigated if there was a difference in gene expression between fibroblasts from control subjects and COPD patients at normoxic conditions and after 4 h of hypoxia exposure. We found that at hypoxia, the mRNA expression of 5-HTR2B (*p* = 0.043, Fig. 1b) and IRE1 (*p* = 0.062, Fig. 1d) was expressed at lower levels in COPD patients compared to control subjects. There was a tendency for increased VEGFR2 expression (*p* = 0.084, Fig. 1a) in fibroblasts from COPD patients compared to control subjects at hypoxic conditions and a tendency for decreased HIF1α (*p* = 0.066, Fig. 1f) in fibroblasts from COPD patients at normoxic conditions compared to control subjects. Several other genes were investigated but the differences were not significantly altered (Additional data 3, Figure Supplement 2).

**Fig. 2:**
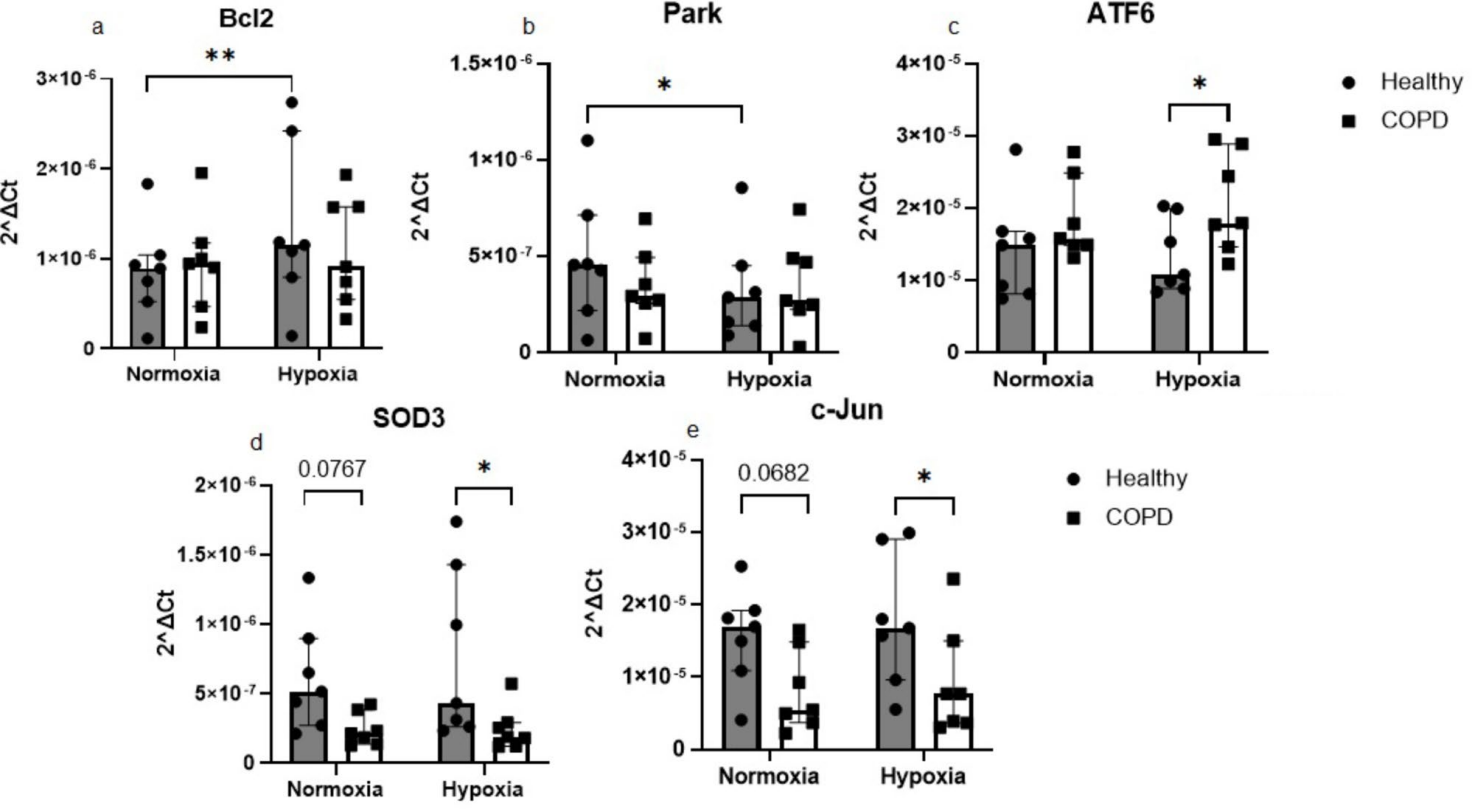
Effects of 24 h of hypoxia on gene expression levels. Genes related to cell death: Bcl2 (**a**), metabolic or mitochondrial stress responses: Park (**b**), cellular stress: IRE1 (**c**) and c-Jun (**e**) and oxidative stress: SOD3 (**d**) were measured by RT-qPCR in primary distal lung fibroblasts obtained from healthy subjects (*n* = 7) and COPD patients (*n* = 7) after 24 h of exposure to normoxic (21% O_2_) or hypoxic (1% O_2_) conditions. Beta-actin and 18 S were used as housekeeping genes. The data is presented as median with interquartile range. Ordinary two-way ANOVA or RM two-way ANOVA were used for unpaired and paired comparisons and the post-hoc test Fisher’s LSD was used for statistical analysis. *p < 0.05, **p < 0.01

Hypoxia exposure during 24 h induced an increased expression of Bcl2 (*p* = 0.007, Fig. 2a) and a decreased expression of Parkin (PARK) (*p* = 0.023, Fig. 2b) in fibroblasts from healthy subjects. When comparing the expression in lung fibroblasts from healthy subjects and COPD patients there was a higher expression of ATF6 (*p* = 0.037, Fig. 2c) and a lower expression of SOD3 (*p* = 0.016, Fig. 2d) and c-Jun (*p* = 0.039, Fig. 2e) in COPD patients at hypoxic conditions. There were also tendencies for lower expression of SOD3 (*p* = 0.077, Fig. 2d) and c-Jun (*p* = 0.068, Fig. 2e) in lung fibroblasts from COPD patients compared to controls at normoxic conditions. Several other genes were investigated but the differences were not significantly altered (Additional data 4, Figure Supplement 3).

### Effects of hypoxia and TGF-β exposure linked to remodelling

**Fig. 3:**
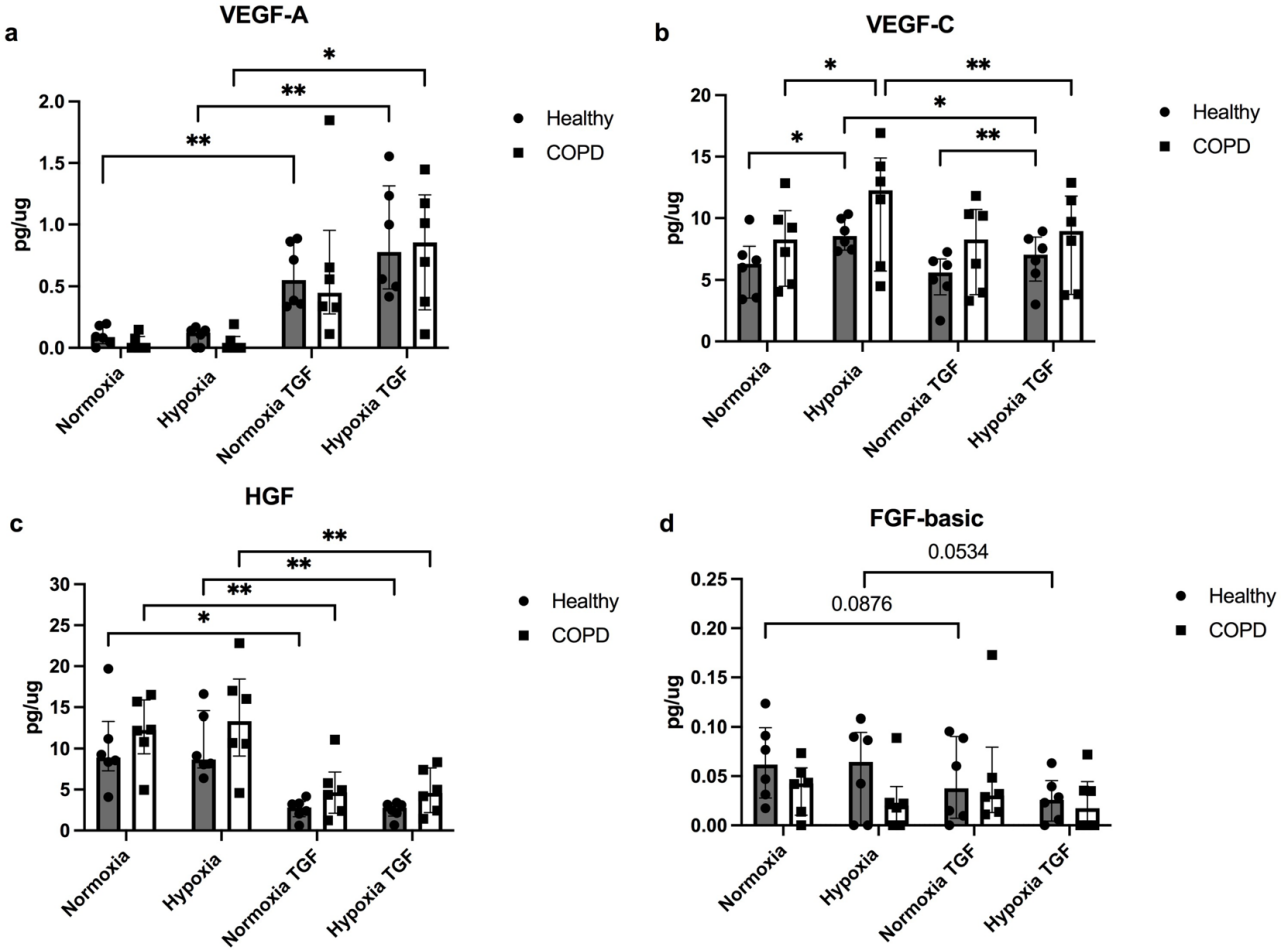
Effects of hypoxia and profibrotic stimuli on mediators linked to remodelling. Release of the following growth factors vascular endothelial growth factors (VEGF)-A (**a**), VEGF-C (**b**), hepatocyte growth factor (HGF), (**c**) and fibroblast growth factor (FGF)-basic (**d**) from lung fibroblasts from healthy subjects (*n* = 6) and COPD patients (*n* = 6) at normoxic (21% O_2_) and hypoxic (1% O_2_) conditions with and without stimuli with transforming growth factor TGF-β1 (10 ng/mL). Total protein levels were used to normalize each sample. The data is presented as median with interquartile range. Ordinary two-way ANOVA or RM two-way ANOVA were used for unpaired and paired comparisons and the post-hoc test Fisher’s LSD was used for statistical analysis. *p < 0.05, **p < 0.01

The levels of several growth factors and inflammatory mediators were investigated after exposure to hypoxia with and without profibrotic stimuli with TGF-β_1_. The levels of VEGF-A were significantly increased after TGF-β exposure in lung fibroblasts from healthy subjects both at normoxic (*p* = 0.0087) and hypoxic (*p* = 0.015) conditions and COPD patients at normoxic (*p* = 0.015) and hypoxic conditions (*p* = 0.0047) (Fig. 3a). There was a tendency for increased VEGF-A in hypoxic condition compared to normoxic conditions after TGF-β stimulation (*p* = 0.076) (Fig. 3a). The levels of VEGF-C were significantly increased after hypoxia in lung fibroblasts from both healthy subjects (*p* = 0.046) and COPD patients (*p* = 0.041) (Fig. 3b), whereas stimuli with TGF-β significantly reduced the response to hypoxia in lung fibroblasts from both healthy subjects (*p* = 0.0323) and COPD patients (*p* = 0.0064) (Fig. 3b). There were decreased levels of HGF in response to TGF-β stimulation in lung fibroblasts from healthy subjects at normoxic (*p* = 0.011) and hypoxic conditions (*p* = 0.0027), and in COPD patients at normoxic (*p* = 0.0027) and hypoxic (*p* = 0.009) conditions (Fig. 3c). Additionally, there was a tendency for decreased levels of FGF-basic after TGF-β stimulation in healthy subjects at normoxic (*p* = 0.088) and hypoxic (*p* = 0.053) conditions (Fig. 3d).

### Effects of hypoxia and TGF-β stimuli linked to inflammation

**Fig. 4:**
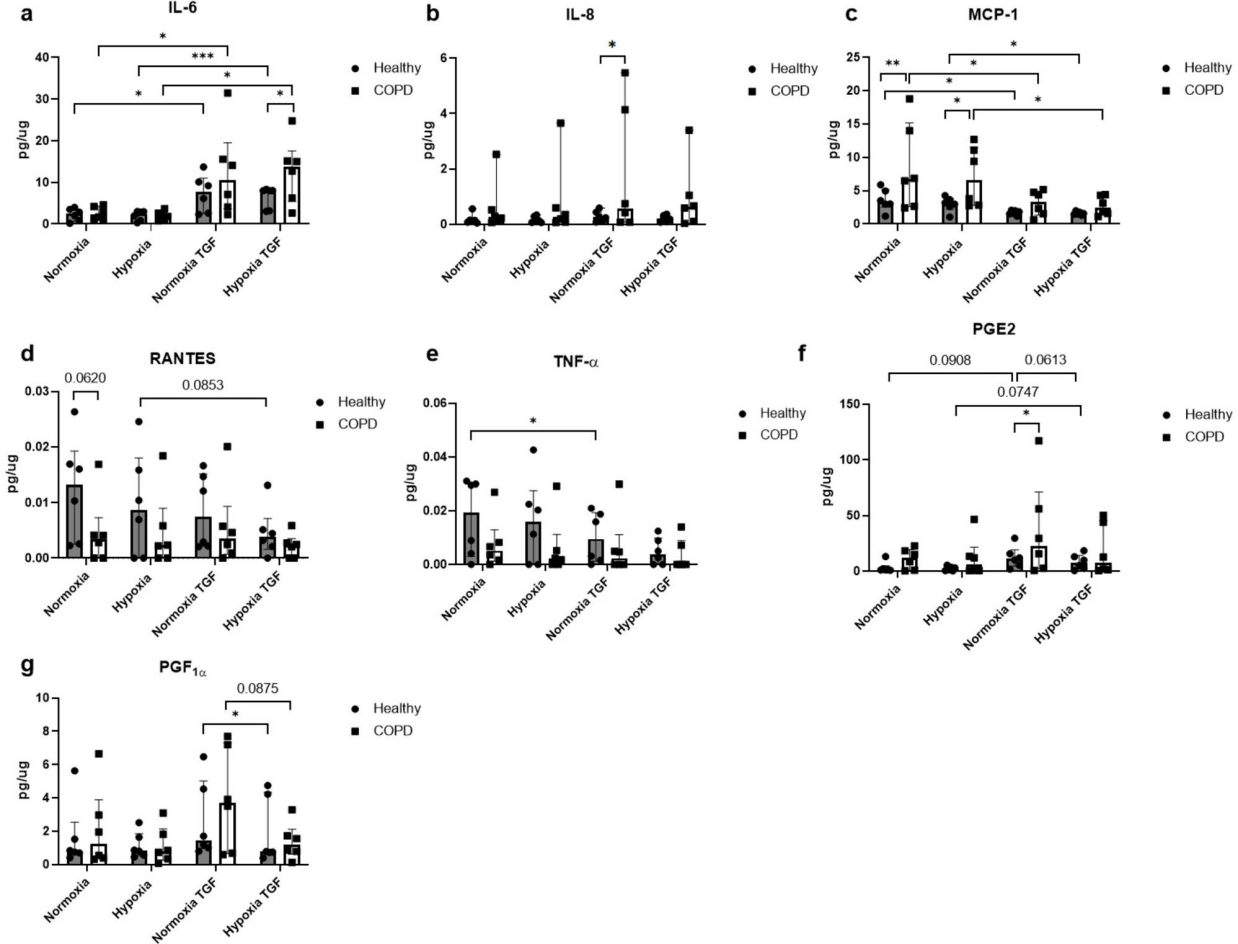
Effects of hypoxia and profibrotic stimuli on inflammatory mediators. Release of inflammatory mediator interleukin (IL)-6 (**a**), IL-8 (**b**), monocyte chemoattractant protein (MCP)-1 (**c**), Regulated upon Activation, Normal T Cell Expressed and Secreted (RANTES) (**d**), Tumor necrosis factor (TNF)-α (**e**), prostaglandin (PG) E_2_ (**f**) and PGF1α (**g**) from lung fibroblasts from healthy subjects (*n* = 6) and COPD patients (*n* = 6) at normoxic (21% O_2_) and hypoxic (1% O_2_) conditions with and without stimuli with transforming growth factor TGF-β1 (10 ng/mL). Total protein levels were used to normalize each sample. The data is presented as mean with min to max. Ordinary two-way ANOVA or RM two-way ANOVA were used for unpaired and paired comparisons and the post-hoc test Fisher’s LSD was used for statistical analysis. *p < 0.05, **:*p* < 0.01, ***:*p* < 0.001

Lung fibroblasts from COPD patients had significantly higher release of MCP-1 (*p* = 0.007,  Fig. 4c), and tendency towards lower release of RANTES (*p* = 0.062,  Fig. 4d) compared to lung fibroblasts from control subjects at basal conditions (normoxia) (Fig. 4). Stimuli with TGF-β induced higher release of IL-8 (*p* = 0.023, Fig. 4b) and PGE_2_ (*p* = 0.036,  Fig. 4f) in lung fibroblasts from COPD patients compared to control subjects at normoxia (Fig. 4). The levels of IL-6 increased after TGF-β stimulation in lung fibroblasts from control subjects at both normoxic (*p* = 0.018) and hypoxic (*p* = 0.0008) conditions, and in lung fibroblasts from COPD patients at both normoxic (*p* = 0.049) and hypoxic (*p* = 0.013) conditions (Fig. 4a). There was a significant higher level of IL-6 in COPD patients compared to healthy subjects at hypoxic conditions after TGF-β stimuli (*p* = 0.039) (Fig. 4a). The levels of MCP-1 were decreased after TGF-β stimulation of lung fibroblasts from control subjects at normoxic conditions (*p* = 0.023) and hypoxic conditions (*p* = 0.030) in fibroblasts from control subjects, and in fibroblasts from COPD patients at normoxic (*p* = 0.048) and hypoxic (*p* = 0.022) conditions (Fig. 4c). The levels of MCP-1 were higher in fibroblasts from COPD patients compared to control subjects at normoxic (*p* = 0.007) and hypoxic (*p* = 0.022) conditions (Fig. 4c). Neither hypoxia nor TGF-β induced any significant alterations in RANTES (Fig. 4d). TNF-α levels were decreased after TGF-β stimulation in lung fibroblasts from healthy subjects at normoxic conditions (*p* = 0.034) and a tendency at hypoxic conditions (*p* = 0.076), whereas there were no effects of either hypoxia or TGF-β stimuli in lung fibroblasts from COPD patients (Fig. 4e). There was a tendency to reduced release of PGE_2_ (*p* = 0.061; Fig. 4f) and a significant decrease in PGF1α levels (*p* = 0.039; Fig. 4g) after hypoxia exposure in TGF-β stimulated fibroblasts from control subjects.

### Expression of HIF2α in lung fibroblasts

**Fig. 5:**
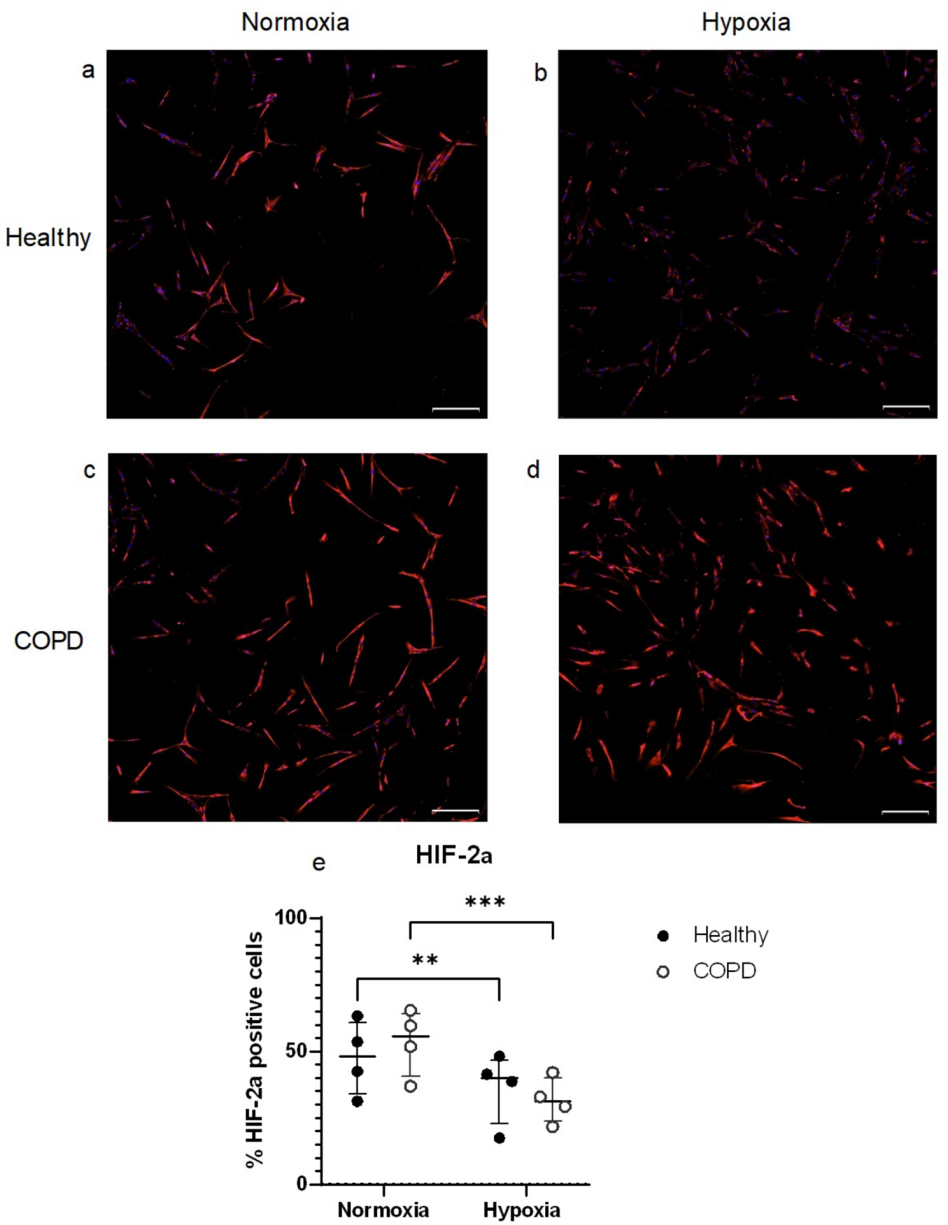
Immunocytochemistry staining and quantification of HIF-2α. Immunofluorescent staining of HIF-2α in distal lung fibroblasts from healthy (**a** and **b**) and COPD (**c** and **d**) subjects at normoxic (**a** and **c**) and hypoxic (**b** and **d**) conditions. The blue stain indicates DAPI and the red stain indicates HIF-2α. Scale bar indicates 200 μm. Quantification of the % positive cells (**e**). Repeated measures ANOVA was used for paired samples and 2-way ANOVA was used for unpaired samples. **p < 0.01, ***p < 0.001

To investigate the effect of 24 h of hypoxia exposure on the expression of HIF2α, the protein expression was analysed using immunocytochemistry (Fig. 5a-e). The HIF2α positive cells were quantified and comparisons were made between healthy (Fig. 5a and b) versus COPD subjects (Fig. 5c and d) at normoxia versus hypoxia. No differences were found between fibroblasts from control and COPD subjects, but when comparing normoxic and hypoxic conditions a downregulation was found at hypoxic conditions both in healthy (*p* = 0.0086) and COPD (*p* = 0.0003) subjects (Fig. 5e).

## Discussion

The overall aims of this study were to investigate if distally-derived lung fibroblasts respond to hypoxic conditions and if there were differences in the response between fibroblasts obtained from healthy subjects and COPD patients. Analyses were performed on mRNA and protein levels. We could detect some differences in RNA expression and secretion of mediators due to hypoxia exposure, although hypoxia did not seem to have profound effects on the fibroblasts. We did also show some differences related to disease pathology, such as differential gene expression profiles and inflammatory pathways between healthy subjects and COPD, and varying responses of fibroblasts to hypoxia between healthy subjects and COPD patients. In general, the present study imply that lung fibroblasts are less responsive to hypoxia exposure compared to epithelial cells, as hypoxia exposure in our previous study on bronchial and alveolar epithelial cells induced several alterations in stress related genes and release of inflammatory mediators and growth factors [14]. These observed differences in response to hypoxia are probably related to the localisation in the lung tissue as mesenchymal cells are not exposed to oxygen in the same degree as the epithelial cells.

### Changes related to oxidative stress and ER stress

We evaluated if hypoxia could affect downstream signalling related to oxidative stress as oxidative stress is linked to COPD pathology [16]. The results show that several genes have a changed expression after exposure to hypoxia primarily in fibroblasts from healthy subjects. After 4 h of hypoxia exposure, fibroblast from healthy subjects showed significantly upregulated expression of IRE1 (UPR), SOD3 (oxidative stress) and c-Jun (cellular stress, apoptosis), while HIF-1α was downregulated. There was a tendency to lower levels of IRE1 after hypoxia exposure in fibroblasts from COPD patients. After 24 h of hypoxia exposure there was a significant higher expression of ATF6 (UPR) and lower expression of SOD3 and c-Jun in fibroblasts from COPD patients compared to healthy subjects. The gene expression data suggests a difference in how lung fibroblasts from healthy compared to COPD patients respond to hypoxia. SOD3 is an extracellular antioxidant and functions as a superoxide anion scavenger limiting inflammation and attenuating oxidative stress by directly binding to and preventing oxidative fragmentation of numerous ECM components, such as type I collagen, heparan sulphate, and hyaluronan [17]. Our obtained results on SOD expression are in line with previous findings in COPD pathology, as a decrease of SOD3 has been related to emphysema. SOD3 attenuated pulmonary emphysema in an in vivo emphysematic mouse models [18] and SOD3 protected against hypoxia-induced pulmonary hypertension in a mouse model with chronic hypoxia [19]. Taken together it has been shown that SOD3 has a protective role both against hypoxia and hallmarks of COPD. We also observed differences in c-Jun which is important for cell proliferation and has anti-apoptotic activity [20]. The gene is regulated by stress stimuli, such as oxidative stress, and it is positively regulated by its own expression [21]. Additionally, c-Jun has been shown to have a role in remodelling and phenotypic shifting in the form of epithelial to mesenchymal transitioning [22]. The reduced expression of SOD3 and c-jun in fibroblasts from COPD subjects after 24 h of hypoxia could indicate that COPD fibroblasts do not proliferate to the same extent and be in a senescent state due to reduced anti-apoptotic signalling, as hypoxia increased Bcl2 gene expression in healthy fibroblasts. It is also possible that there is reduced abilities of phenotypic shifting in COPD fibroblasts which could contribute to the remodelling present in COPD [22].

Transcription factor Nrf2 regulates expression of antioxidant genes, and reduced levels were present in COPD lungs [23]. Nrf2 showed protective effects by inducing antioxidant and antiprotease gene transcription in macrophages, but again, a decreased expression of Nrf2 was present in smokers with COPD [24]. However, we could not detect any significant differences in Nrf2 gene expression in lung fibroblasts. Hypoxia has been shown to increase proteostasis in COPD patients [25, 26] and induce related stress pathways [27]. PINK1 is a kinase that phosphorylates proteins and regulates activity of Parkin (PARK) [28]. In the present study fibroblasts from healthy subjects showed a significant decrease of PARK. Parkin is a ubiquitin ligase involved in protection from mitochondrial dysfunction (mitophagy) by labelling proteins for proteasomal degradation in cellular stress responses such as unfolded proteins [29], but the function of its gene is not completely understood. ER stress results in un- and misfolded proteins often caused by oxidative stress. Lungs of smokers and COPD patients experience enhanced oxidative stress [2]. Our obtained data indicates that there is a difference in the hypoxic ER stress response (IRE1 and ATF6) between lung fibroblasts from healthy subjects and COPD patients. In a previous study in mouse fibroblasts, hypoxia induced an unfolded protein response by PERK activation, independent on HIF-1α activation [30]. Weidner et al. showed disrupted ER, Golgi and lysosomal structures in fibroblasts derived from COPD patients compared to healthy individuals [10]. Altogether, our current findings on changes in gene expression related to oxidative stress and ER stress need further investigations.

### Changes related to remodelling

Airway and pulmonary vascular remodelling are processes associated with COPD pathogenesis and severity where hypoxia and hypoxaemia has been shown to play an important role in these remodelling processes [4, 31]. Pulmonary hypertension (PH) is very common in endstage COPD) [31, 32]. PH is suggested to be the result of hypoxia associated with COPD and vascular alterations have been shown to often appear before emphysema is even detectable [33]. In the current study, differences related to remodelling as alterations in collagen7, 5HTR2B and VEGF were seen between healthy and COPD fibroblasts. Collagen VII is a crucial basal membrane protein that forms anchoring fibrils and turnover rate of type VII collagen was significantly increased in serum samples from COPD patients [11]. Our results show that levels of Collagen7 mRNA were affected by hypoxia in lung fibroblasts from healthy subjects, where an increase was seen after 4 h and a decrease in 24 h exposure to hypoxia, however, no changes were seen in fibroblasts from COPD patients. 5-HTR2B mRNA levels were significantly increased after 4 h hypoxia exposure in lung fibroblasts from healthy subjects and the expression levels of 5-HTR2B mRNA were lower in COPD patients compared to healthy subjects. 5-HTR2B has previously been shown to be upregulated in pulmonary fibrosis [12, 34], suggesting that the opposite may occur in COPD. Our results showed very low expression of VEGFRs on protein and mRNA level in fibroblasts, which seemed to be differently affected by hypoxia in disease. Basal VEGFR2 mRNA levels appeared to be higher in COPD patients compared to healthy. Previous immunocytochemistry staining has shown that distally derived lung fibroblasts from healthy individuals and patients with GOLD IV express all three VEGFRs [6]. VEGFR1 and VEGFR2 have predominantly been reported to be expressed by endothelial cells, while VEGFR3 is expressed on lymph vessels. In these cell types, all receptors were shown to be upregulated after hypoxia [35]. Studies also reported an increase of VEGF and VEGFR2 expression in COPD that was positively correlated with HIF-1α and associated with disease severity [3]. Another study showed that the response to hypoxia relating HIF-1 and subsequent VEGF expression was lower in COPD patients compared to healthy controls (non-smokers and smokers) [36]. However, our obtained data show that VEGF-C, but not VEGF-A, is increased by hypoxia in the fibroblasts (both in healthy and diseased). Our data show an increase of VEGF-A, but not VEGF-C, in fibroblasts in response to TGF-β1, whereas the hypoxia-induced release of VEGF-C was decreased by TGF-β, indicating that the VEGF isoforms are regulated in different ways in the fibroblasts. Upregulation of both VEGF-A and VEGF-C expression has been detected in lung donor grafts [37]. VEGF-C is important for lymphangiogenesis but also involved in vascular remodelling and could be a potential biomarker as VEGF can be measured in serum samples. The role of VEGF-C in different stages and phenotypes of COPD is however less described and remains to be further explored. Morfoisse et al. has previously shown in a tumour model that VEGF-C expression was increased during hypoxic conditions, however the transcription was not induced via HIF-1α [38]. In our current study profibrotic stimuli with TGF-β decreased response to hypoxia in both VEGF-C and HGF levels in lung fibroblasts from both COPD patients and healthy subjects. HGF have shown anti-apoptotic effects on epithelial and endothelial cells [39] and that HGF induces apoptosis and prevents accumulation of myofibroblasts in experimental lung fibrosis, while in vitro studies have shown that HGF is involved in the regulation of TGF-β and inhibits epithelial-mesenchymal transitions [40]. Both VEGF and HGF levels have been shown to be reduced in COPD patients, which may further contribute to emphysema in the lung parenchyma [41].

### Changes related to inflammation

The inflammatory markers IL-6, IL-8 and MCP-1 are recognised as drivers in pulmonary fibrosis but also in COPD pathology [42]. In the present study, MCP-1 was the only cytokine that was significantly increased in fibroblasts from COPD patients, whereas there was a tendency towards lower release of RANTES at normoxia (basal conditions). Hypoxia had in general no effect on the release of inflammatory mediators. Stimuli with TGF-β induced a significantly increased release of IL-8 and PGE_2_ in COPD fibroblasts compared to healthy fibroblasts, whereas increased IL-6 and decreased MCP-1 levels were observed in both COPD and healthy fibroblasts. Hypoxia exposure alone without TGFβ stimuli had in general no significant effect on the release of inflammatory cytokines. The combination of hypoxia and TGF-β induced significantly higher release of IL-6 in COPD fibroblasts compared to healthy fibroblasts. In our previous study on epithelial cells, hypoxia and TGF-β_1_ stimuli increased the release of IL-6 and MCP-1 in bronchial epithelial cells and IL-6 in alveolar epithelial cells [14]. MCP-1 plays a role in antiviral immune response and regulates infiltration and migration of innate immune cells. MCP-1 has been found to be increased in sputum [43] and blood from COPD subjects [44]. This matches the increased levels of MCP-1 found in our study in lung fibroblasts from COPD subjects and is logical since one of the characteristics of COPD is inflammation. Previous studies in endothelial cells have seen an increased release of IL-6 and IL-8 in response to hypoxia [45]. Both IL-6 and IL-8 have been shown to be increased in fibroblasts from COPD patients [46]. These findings overlap partly with our obtained data where we found higher levels in COPD fibroblasts, but only after profibrotic stimuli. Both IL-6 and IL-8 are cytokines with multiple proinflammatory functions and an increased level of these are expected in inflammatory diseases such as COPD. The response to profibrotic stimuli was however similar in lung fibroblasts from control and COPD subjects., indicating that the response to TGF-β is not impaired in COPD, though the profibrotic stimuli had the potential to induce or exasperate differences both between healthy and COPD (as seen in IL-6, IL-8, and PGE_2_), and between normoxia and hypoxia (as seen in PGF_1α_). As hypoxia had limited effects on the release of inflammatory markers there is a possibility that some of these markers need more than one kind of stimuli to be induced in lung fibroblasts. RANTES is a chemokine that attracts several different kinds of immune cells. There is only limited literature on RANTES in COPD, but it has been shown to be increased during viral induced exacerbations [47]. In this study we have seen a tendency for lower levels of RANTES in lung fibroblasts from COPD patients, which is in contrast to what has been found in sputum, where no difference could be found [48]. The difference between the results could be explained by the different cell types investigated and in vitro versus in vivo conditions. Altogether, this data indicate that hypoxia may aggravate inflammatory responses that are already present in COPD patients.

### Expression of HIF-2α

Immunocytochemistry staining of lung fibroblasts have revealed the presence of HIF-2α both at normoxia and hypoxia in lung fibroblasts from both healthy and COPD subjects. This is, to our knowledge, the first time HIF-2α expression has been investigated in hypoxia stimulated distal lung fibroblasts from human subjects. In this study we have found the expression of HIF-2α to be decreased after 24 h hypoxia (1% O_2_). This is in contrast to human lung and pulmonary artery adventitial fibroblasts which have been shown to have an upregulation of HIF-2α under similar conditions [49, 50]. This disparity could be due to differences between central vs. distally derived fibroblasts [13]. In a previous study in human alveolar epithelial cells we also detected an upregulation of HIF-2α after exposure to hypoxia (1% O_2_) [14]. It has been shown that the expression levels of HIF-2α is different in different cell types and different organs in rats [51] which also may be the case in the human lung. The same study also found a downregulation of HIF-2α protein in the lung after 12 h. This is in line with what we have found in this study and indicates that the HIF-2α response in the lung probably occurs at an earlier timepoint than in other tissues. This could be due to the lung being the main organ for oxygen exchange and thereby more sensitive to hypoxia. Additionally, HIF-2α has been shown to regulate genes that are also regulated by HIF-1α [52]. Mizuno et al. have shown lower levels of HIF-1α and VEGF in patients with severe COPD, classified by the Global Initiative for COPD [53], indicating impairment of lung maintenance programs. In our study we did not found statistical differences in the expression of HIF-2α between lung fibroblasts from healthy and COPD subjects, which could indicate that this pathway is not impaired in fibroblasts from COPD subjects, but further research in the area is warranted.

#### Strengths and limitations of the study

Fibroblasts have often been studied in association to disease pathogenesis related to remodelling, however not much is known about these cells in COPD patients. As there is limited data published on hypoxia and fibroblasts and especially distally derived lung fibroblasts from COPD patients, we were intrigued how this cell type would respond to hypoxia exposure, mimicking on episode which could occur during an exacerbation with increased airflow limitations, and if there would be differences in response between healthy and COPD. This study used primary human material, which is more representative of real disease than cell lines, as it includes heterogeneity between patient groups and individuals. However, this study investigated a limited number of patients with moderate or very severe COPD to compare potential differences between disease stages of COPD. As this study provided some interesting findings, future studies should include more individuals within the different GOLD stages. In the current study we analysed gene expression after 4 h and both gene expression and protein levels after 24 h. Alterations in gene expression could have appeared at other time points that we were not able to detect due to unstable or degraded mRNA. Furthermore, it would be interesting to investigate whether some anti-fibrotic or anti-angiogenic drugs that act on VEGF signalling pathways could induce responses of healthy fibroblasts in COPD patients upon treatment, or vice versa, to model COPD.

## Conclusions

Our obtained data in this study provide insights into different expression profiles associated with stress responses and remodelling processes during hypoxic and normoxic conditions, as alterations in gene expression of c-jun, SOD3, IRE, 5HTR2B, VEGFR2 and collagen7 in fibroblasts from control subjects and COPD patients. Our primary findings indicate decreased response to hypoxia in regulation of 5-HTR2B and IRE1 gene expression, increased release of MCP-1 and VEGF-C in COPD fibroblasts and that profibrotic stimuli resulted in higher IL-8 and PGE_2_ release in COPD fibroblasts. These results give information about altered responses in lung fibroblasts from COPD patients and may contribute towards improved understanding of the heterogeneity of COPD pathology. Our findings indicate that hypoxia and profibrotic stimuli induced cellular responses with altered inflammatory profile with fibroblasts from COPD patients being less responsive to hypoxia compared to control subjects. Since both hypoxia and inflammation are common in the disease this constant environment of stress could desensitize the lung fibroblasts to external stimuli.

### Electronic supplementary material

Below is the link to the electronic supplementary material.

Supplementary Material 1

Supplementary Material 2

Supplementary Material 3

Supplementary Material 4

## Data Availability

The data will be supplied upon request to corresponding authors.

## Abbreviations

ATF6: Cyclic AMP-dependent transcription factor ATF-6 alpha
c-Jun: Transcription factor AP-1 oncogene
CHOP: DNA damage-inducible transcript 3 protein
COPD: Chronic obstructive pulmonary disease
ECM: Extracellular matrix
FGF-2: Fibroblast growth factor-basic
HGF: Hepatocyte growth factor
HIF: Hypoxia-inducible factor
IL: Interleukin
IRE1: Serine/threonine-protein kinase/endoribonuclease
MCP-1: Monocyte chemotactic protein-1
NRF2: Nuclear factor erythroid 2-related factor 2
OXR1: Oxidation resistance protein 1
PERK: Eukaryotic translation initiation factor 2-alpha kinase 3
PGE2: Prostaglandin E2
PINK1: Serine/threonine-protein kinase
PSMA1: Proteasome subunit alpha type-1
PSMB6: Proteasome subunit beta type-6
PSMD11: 26 S proteasome non-ATPase regulatory subunit 11
PTGS2: Prostaglandin G/H synthase 2
RANTES: Regulated upon Activation, Normal T Cell Expressed and Secreted
SOD3: Extracellular superoxide dismutase
TGF-β1: Transforming growth factor-beta 1
TNF: Tumour necrosis factor
UPR: Unfolded protein response
VEGF: Vascular endothelial factor
WST-1: Water-soluble tetrazolium salt 1
