# Supplementary material for: Altered hypoxia-induced cellular responses and inflammatory profile in lung fibroblasts from COPD patients compared to control subjects
Source: Respir Res. 2024 Jul 16;25:282. doi: 10.1186/s12931-024-02907-x (PMC11253402; doi:10.1186/s12931-024-02907-x)
Supplement: Supplementary file 1 — Supplementary Material 1 [file 12931_2024_2907_MOESM1_ESM.docx]

Table S1. Genes of interest analysed in RT-qPCR, including primer sequences for 5’ and 3’ gene sequences.

| **Gene** | Forward primer (5’) | Reverse primer (3’) |
| --- | --- | --- |
| **GAPDH** | GAA GGT GAA GGT CGG AGT CA | TGG AAG ATG GTG ATG GGA TT |
| **b-actin** | AGC ACA GAG CTT CGC CTT T | GGA ATC CTT CTG ACC CAT GC |
| **CHOP** | GAT TCC AGT CAG AGC TCC CT | GCC TCT ACT TCC CTG GTC AG |
| **NRF2** | CAC TTG TTC CTG ATA TTC CCG G | ATA GCT CCT CCC AAA CTT GCT |
| **IRE1** | AGA TGC ACC AAG TAC AGC CT | CCT AAT GCC ACA CCT CAT GC |
| **PERK** | TCA CAG GCA AAG GAA GGA GT | AAC AAC TCC AAA GCC ACC AC |
| **ATF6** | GCT CTC TTT GCT GAA CTC GG | TGA GGA GGC TGG AGA AAG TG |
| **PSMA1** | AGG GCA GGA TTC ATC AAA TTG A | AAG CCC CGC AAT TGA GAT AC |
| **OXR1** | AGT GTT GAT TGC CAG GTT GC | GAC AAG GAA ATG ATG CCC TGT |
| **Bcl2** | TGC TGA AGA TTG ATG GGA TCG | TCA CGC GGA ACA CTT GAT TC |
| **VEGFR1** | CAA ATA AGC ACA CCA CGC CC | TGC TTT GGT CAA TTC GTC GC |
| **VEGFR2** | AGT CTG TGG CAT CTG AAG GC | CCG AGT CAG GCT GGA GAA TC |
| **VEGFR3** | GAG TTC CTG GCT TCC CGA AA | AGA TGC TTT CAG GGG CCA TC |
| **5-HTR2B** | CTC ACT GGC TGC CTT CTT CA | CCA GCA TTG CCA CCT TTT CC |
| **Collagen 7** | GTG AGG ACT GCC CCT GAG | GAC TCC ACC TTC GAG ACC C |
| **PSMB6** | GAA TCA TCA TCG CAG GCT GG | TGC AGA CAC TCT TCC TTG GT |
| **PSMD11** | TGG GGT GTG GTT TCT CTC TC | CCT CGC TGT GTA ATC GTG TC |
| **HIF1a** | CCC ATT TTC TAC TCA GGA CAC AG | CTG ATC GAA GGA ACG TAA CTG G |
| **Parkin** | GCA GAG ACC GTG GAG AAA AG | TCC TTC CTG CTG TCA GTG TG |
| **PINK1** | CCA GGC AAT TTT TAC CCA GA | AAT GTA GGC ATG GTG GCT TC |
| **18S** | AAA CGG CTA CCA CAT CCA AG | CCT CCA ATG GAT CCT CGT TA |
| **SOD3** | GAG ATC TGG CAG GAG GTC AT | GAA CTG GTG CAC GTG GAT G |
| **c-Jun** | CCC CAA GAT CCT GAA ACA GA | CCG TTG CTG GAC TGG ATT AT |
| **PTGS2** | ATC ACA GGC TTC CAT TGA CC | CAG GAT ACA GCT CCA CAG CA |
