# Supplementary material for: Altered hypoxia-induced cellular responses and inflammatory profile in lung fibroblasts from COPD patients compared to control subjects
Source: Respir Res. 2024 Jul 16;25:282. doi: 10.1186/s12931-024-02907-x (PMC11253402; doi:10.1186/s12931-024-02907-x)
Supplement: Supplementary file 2 — Supplementary Material 2 [file 12931_2024_2907_MOESM2_ESM.docx]

**Additional data 2**

***Results:* *Experimental settings of hypoxic conditions in healthy primary lung fibroblasts***

A pilot study with different oxygen concentrations (1 %, 2 % or 5 % O_2_), serum concentrations (0.4 %, 1 % or 2 %) in the culture medium and time points (4 h, 24 h, 48 h and 72 h) were evaluated for the settings for hypoxia exposures in primary healthy lung fibroblasts (S1a-m). None of the tested serum concentrations or time settings increased LDH release (figure S1a-c) or metabolic activity in hypoxic culture conditions, except for increased metabolic activity at 2 % serum (figure S1d-f). Evaluation of HIF-1α indicated that exposure to 1 % O_2_ increased mRNA levels of HIF-1α (figure S1g). Increased mRNA levels of HIF-1α was observed in primary healthy lung fibroblasts cultured with 0.4 % serum in 4 h of hypoxia, whereas the expression was reduced after 24 h of hypoxia. Higher serum content (1 % or 2 %) did not alter mRNA levels of HIF-1α at hypoxia. HIF-1α was undetected or showed very low expression levels independent of serum concentrations after 72 hours (figure S1h). The genes Nrf2 (marker for oxidative stress), 5HTR2B (involved in tissue remodelling) and VEGFR2 (involved in angiogenesis) showed higher gene expression after hypoxia exposure (1% O_2_) at 24 h compared to 4 h and 72 h. No differences were observed in gene expression for Bcl2 (apoptosis) and VEGFR1 (involved in angiogenesis). VEGFR3 (angiogenesis/lymphoangiogenesis) was not measurable at 4 h and 24 h but detected after 72 h in both normoxia and hypoxia (figure S1i). The expression of 5HTR2B and HIF-1α was increased in fibroblasts cultured in hypoxia (figure S1k and m) compared to normoxia (figure S1j and S1l). Based on these experiments, we set hypoxia exposure to 1% oxygen, exposure time to 4 h and 24 hours and cell medium containing 0.4% serum for following experiments with primary lung fibroblasts.


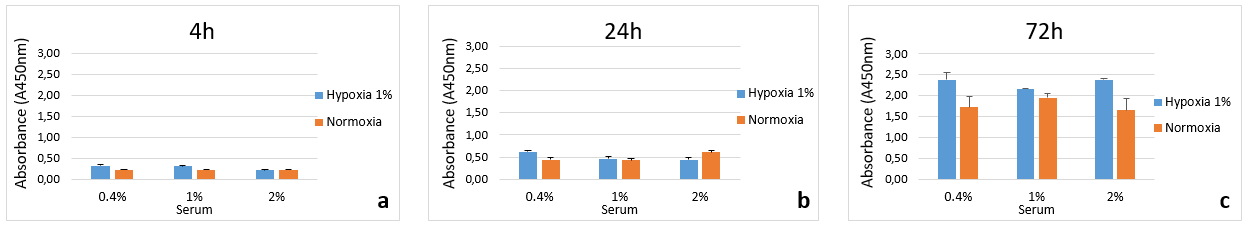

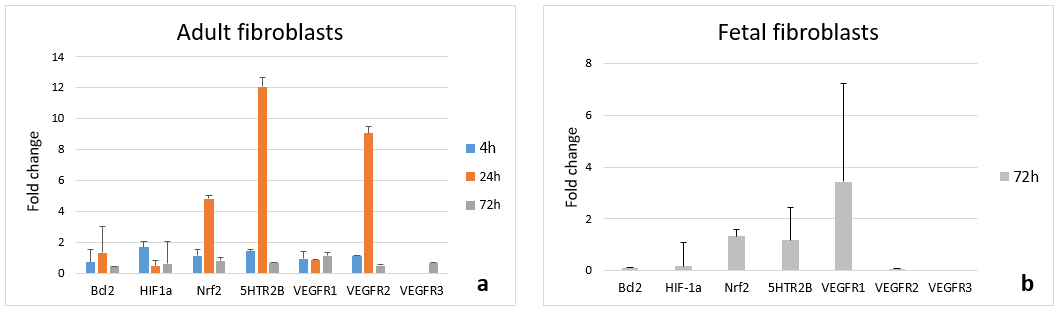

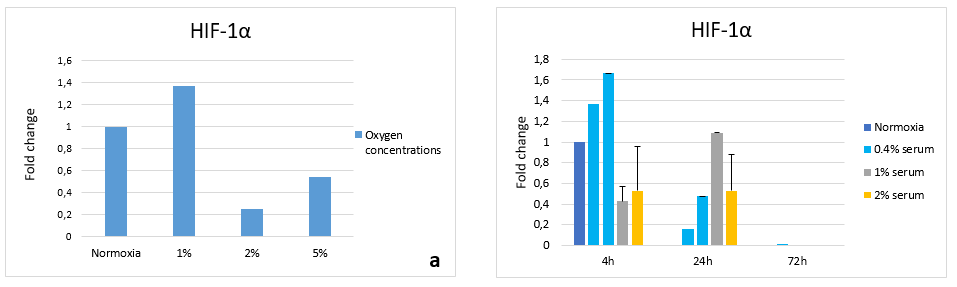

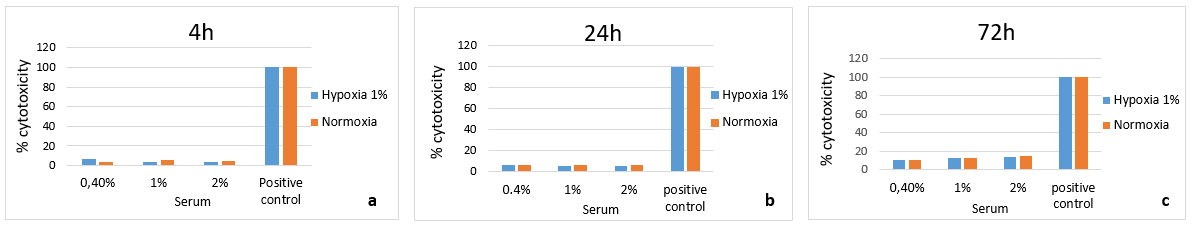


**a b c**

**d e f**

**g h i**


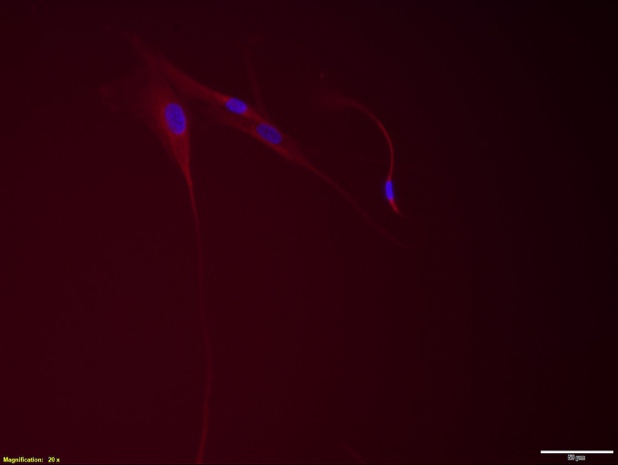

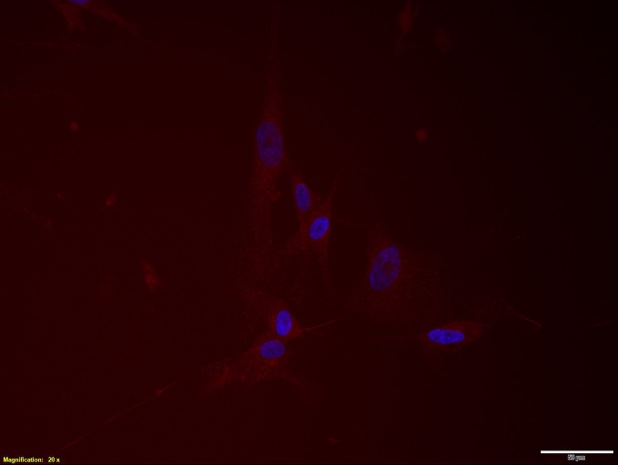

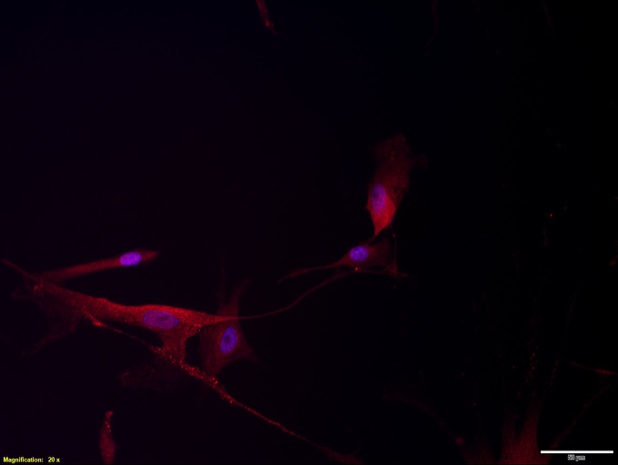

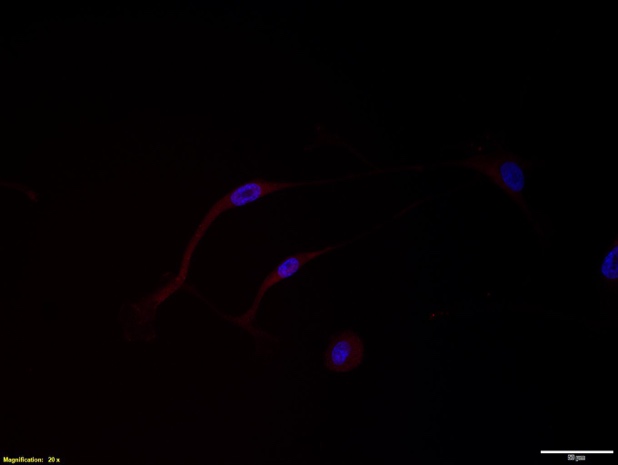


**j**

**k**

**l**

**m**

***Figure S1.*** *Cytotoxicity, measured as LDH release compared to positive control Triton x100 (2%) (a-c) and metabolic activity (d-f), in primary distal derived lung fibroblasts obtained from a healthy control and cultured with different serum concentrations 0.4 %, 1 % and 2 % in either normoxic or hypoxic conditions for 4 h (****a and d****), 24 h (****b and e****) and 72 h* ***(c and f****). Red bars indicate normoxia (21 % oxygen) and blue bars hypoxia (1 % oxygen). n=2 for all samples and the bars represent mean values +/- S.D of the two replicates. Expression of HIF-1α gene expression in fibroblasts cultured with medium containing 0.4% serum in different oxygen concentrations (normoxia (21%), 1%, 2% and 5%) for 4 h (g) and at different time points (4 h, 24 h and 72 h) with 1% oxygen in different serum concentrations (0.4 %, 1 % and 2 % serum) (h). All genes are normalized to respective control in normoxia. n=two technical replicates for each sample and the bars indicate mean +/- S.D. There were two different measurements for 0.4% serum and therefore two bars in each time interval (h).* Gene expression for Bcl2, HIF-1a, 5HTR2B, VEGFR1, VEGFR2 and VEGFR3 in l*ung fibroblasts cultured in 4 h (blue bars), 24 h (red bars) and 72 h (grey bars) with 18S as housekeeping gene (figure* *i). Antibody labelling of serotonin receptor 2B (5HTR2B) and HIF-1α using rabbit monoclonal antibodies with magnification x20. The cell nuclei were stained with DAPI (blue) and the serotonin 2B receptors (j and k) and HIF-1a (l and m) are shown in red. Fibroblasts* were *cultured in either normoxia (21% oxygen, 0.4% serum, 24 h) (fig. j and l) or hypoxia (1% oxygen, 0.4% serum, 24 h) (figure k and m). Scale bar indicates 50 µm.*
