# Supplementary material for: Altered hypoxia-induced cellular responses and inflammatory profile in lung fibroblasts from COPD patients compared to control subjects
Source: Respir Res. 2024 Jul 16;25:282. doi: 10.1186/s12931-024-02907-x (PMC11253402; doi:10.1186/s12931-024-02907-x)
Supplement: Supplementary file 4 — Supplementary Material 4 [file 12931_2024_2907_MOESM4_ESM.docx]

***
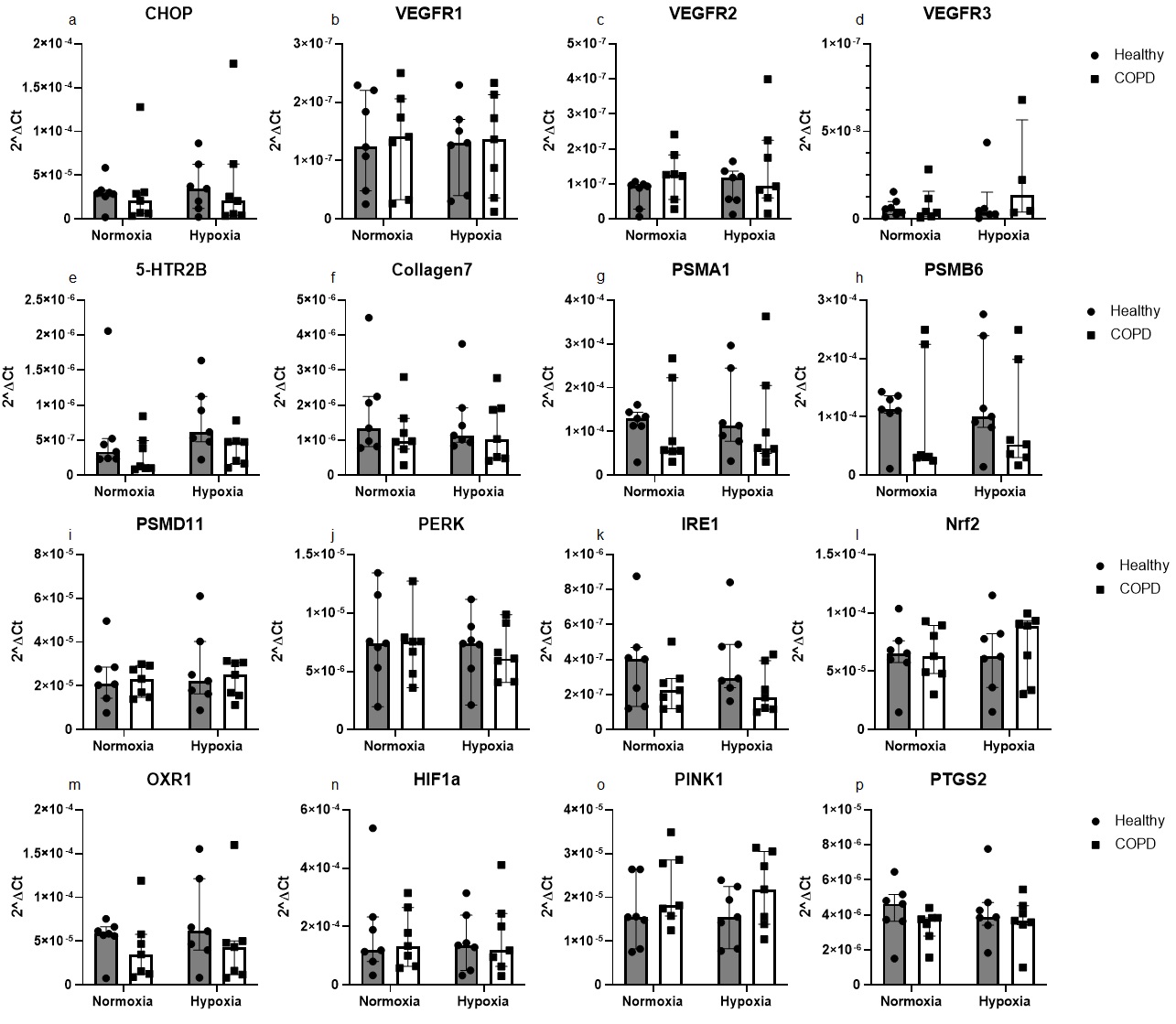
*Additional data 4**

***Figure S3.*** ***Effect of 24 hours of hypoxia exposure on gene expression levels****.* Genes where no statistically significant differences were found in the mRNA measured with RT-qPCR in primary distal lung fibroblasts obtained from healthy subjects (n=7) and COPD patients (n=7) after 24 hours of exposure to normoxic (21% O_2_) or hypoxic (1% O_2_) conditions. Geomean of beta-actin and 18S were used as housekeeping. The data is presented as median with interquartile range. Ordinary two-way ANOVA or RM two-way ANOVA were used for unpaired and paired comparisons and the post-hoc test Fisher’s LSD was used for statistical analysis.
